# What is the role of video laryngoscopy in pre-hospital care?

**DOI:** 10.1186/1757-7241-22-S1-A6

**Published:** 2014-07-07

**Authors:** Wolfgang G Voelckel

**Affiliations:** 1 Department of Anaesthesiology and Critical Care Medicine, AUVA Trauma Centre Salzburg, Austria; 2 ÖAMTC Air Rescue, Vienna, Austria

## Background

Direct laryngoscopy is the primary method for performing tracheal intubation in the pre-hospital setting. Failure to quickly establish an airway can result in hypoxemia, aspiration, neurologic damage, cardiovascular complications, and death. In-hospital data derived form 50,760 apparently normal patients undergoing surgery suggest that difficult laryngoscopy occurs in 5.8% [1]. When intubation is required in a pre-hospital scenario, the incidence of difficult laryngoscopy is doubled even when experienced anaesthesiologists are involved, and expected to reach some 20% when less qualified operators are in charge [2]. Thus, failed intubation rates in the emergency medical service may be as high as 7% [3].

## Technique

Videolaryngoscopy is a relatively new technology developed to improve the success rate of tracheal intubation. A high-resolution micro camera mounted on the tip of a curved blade connected to small portable digital monitor improves the view of the vocal cords and subsequently the success rate of direct laryngoscopy. During direct laryngoscopy, the operator visualizes the larynx from outside the oral orifice. The distance between the vocal cords and the laryngoscopist’s eye is significant (30–40 cm). This reduces the angle of view to some 15° with a classic laryngoscope. Suboptimal alignment of the oral-, pharyngeal- and laryngeal-axis, as well as multiple anatomic orpathologic factors can make direct visualization of the airway difficult or even impossible. Videolaryngoscopy significantly widens the angle of view since the digital camera and light source are inserted very close (2–3 cm) to the larynx.

**Figure 1:**
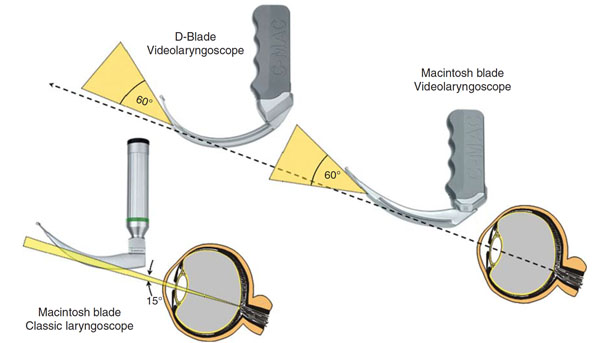
Van Zundert A *BJA* 2012

Videolaryngoscopes or alternative optic devices providing a “view around the corner” may be categorized according to design, handling and guidance of the tracheal tube.

**Figure 2:**
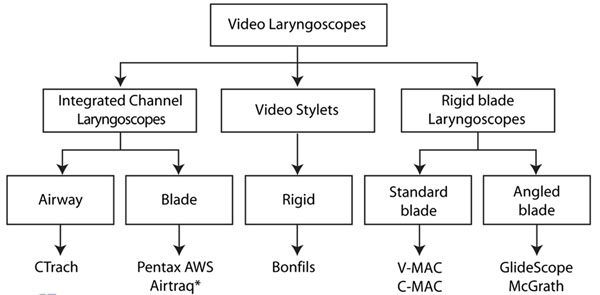
Healy D *Anesthesiology* 2012

## General considerations

There is sufficient scientific evidence that videolaryngoscopy improves the visualization of the vocal cord when established scoring systems such as the Cormack and Lehane scale are employed. It is noteworthy that despite an improvement in view, the operator may still be unable to pass a tracheal tube through the glottis. Thus, the key to a successful outcome is not solely due the view obtained but related to the ease of inserting the tracheal tube. This must be considered in future studies evaluating the role of videolaryngoscopy in the pre-hospital setting. In this regard, a three part scoring system comprising the device employed, the view (full, partial, none) and the ease of intubation (easy, modified, unachievable) has been suggested [4]. In addition, performance of videolaryngoscopy in patients endangered by mucus, blood or vomitus in the upper airway must be addressed. Finally, videolaryngoscopy will be challenged by typical pre-hospital environmental obstacles, i.e. bright ambient light, humidity, rain, snowfall, extreme cold or heat. In this regard, a robust and reliable device is key when used outside the hospital.

## Learning curve

Similar to the learning curve for direct laryngoscopy, new airway devices require significant training and experience. Manikin studies do not allow a comprehensive appreciation of the training needed. At present, available studies suggest a fairly low number of intubations needed to obtain the required skills [5].

## Scientific evidence

Studies and case reports suggest that intubation success rates may be as high as 99% when direct laryngoscopy was impossible [6]. However, in a systematic review addressing the potential role of videolaryngoscopy in successful orotracheal intubation [7], the evidence found for efficacy in difficult airway management was limited (Table 1).

**Table 1:** 

	Good evidence (Level 1+)	Weak evidence (Level 3)	No evidence
**Subjects at higher risk of difficulty during DL**	Airtraq	Bonfils	McGrath
	CTrach	Bullard	
	GlideScope		
	Pentax AWS		
	V-MAC		
**Known difficult DL**		Airtraq	McGrath
		Bonfils	V-MAC
		Bullard	
		CTrach	
		GlideScope	
		Pentax AWS	
**Failed DL**		Airtraq	Bullard
		Bonfils	V-MAC
		CTrach	
		GlideScope	
		McGrath	
		Pentax AWS	

## Evidence obtained in pre-hospital studies

The number of randomized control trials (RCT) addressing videolaryngoscopy in the pre-hospital setting is still low. Presently, there is only one RCT comparing direct laryngoscopy (DL) with the AirTraq in 212 patients. Success rates for DL were found to be 98% whereas the AIrTraq intubation success was as low as 47.2% [8]. The authors concluded that besides some technical shortcomings, pre-study training was insufficient, although operators felt confident using the device. Preliminary reports and prospective observational studies addressing the C-Mac videolaryngoscope in helicopter [9,10] and ground emergency medical services [11] found the device suitable and useful for prehospital emergency tracheal intubations with complicated airway conditions.

## Conclusion

Presently, the data available does not or insufficiently address the specific aspects of intubation in the exposed pre-hospital environment. In addition, some typical problems such as on-going bleeding or vomitus in the upper airway have still not been evaluated in pre-hospital RCTs. Since the C-Mac videolaryngoscope (K. Storz, Tuttlingen Germany) combines a conventional designed laryngoscope with video imaging, this concepts merits appreciation and may already be implemented in field care.

In conclusion, videolaryngoscopy is a rapidly growing technology. In the future, video laryngoscopy will dominate the field of emergency airway management.

## Competing interests

The author declares no conflict of interest with the devices or the technology addressed in this summary.
